# The Cdc42 Effector Kinase PAK4 Localizes to Cell-Cell Junctions and Contributes to Establishing Cell Polarity

**DOI:** 10.1371/journal.pone.0129634

**Published:** 2015-06-11

**Authors:** Widyawilis Selamat, Pei-Ling Felicia Tay, Yohendran Baskaran, Ed Manser

**Affiliations:** 1 small G-protein Signaling and Kinases (sGSK) Group, Institute of Molecular and Cell Biology, Agency for Science, Technology and Research (A*STAR), Singapore, Singapore; 2 Institute of Medical Biology, Agency for Science, Technology and Research (A*STAR), Singapore, Singapore; 3 Department of Pharmacology, National University of Singapore, Singapore, Singapore; The Beatson Institute for Cancer Research, UNITED KINGDOM

## Abstract

The serine/threonine kinase PAK4 is a Cdc42 effector whose role is not well understood; overexpression of PAK4 has been associated with some cancers, and there are reports that correlate kinase level with increased cell migration *in vitro*. Here we report that PAK4 is primarily associated with cell-cell junctions in all the cell lines we tested, and fails to accumulate at focal adhesions or at the leading edge of migrating cells. In U2OS osteosarcoma and MCF-7 breast cancer cell lines, PAK4 depletion did not affect collective cell migration, but affected cell polarization. By contrast, Cdc42 depletion (as reported by many studies) caused a strong defect in junctional assembly in multiple cells lines. We also report that the depletion of PAK4 protein or treatment of cells with the PAK4 inhibitor PF-3758309 can lead to defects in centrosome reorientation (polarization) after cell monolayer wounding. These experiments are consistent with PAK4 forming part of a conserved cell-cell junctional polarity Cdc42 complex. We also confirm β-catenin as a target for PAK4 in these cells. Treatment of cells with PF-3758309 caused inhibition of β-catenin Ser-675 phosphorylation, which is located predominantly at cell-cell junctions.

## Introduction

Mammalian PAK isoforms are categorized into two groups on the basis of their structural and biochemical features: conventional or group I PAKs comprise PAK1-3, and the group II PAKs (PAK4-6) are encoded by three genes in mammals [1–3]. The group II PAKs are predominantly mammalian effectors of Cdc42 with PAK4 being a ubiquitously expressed essential kinase [4,5]. PAK4-null mice are embryonic lethal [6] due to defects in the fetal heart as well as in neuronal development and axonal outgrowth, while PAK5-/PAK6-null mice exhibit only neuronal defects [7]. A number of studies have shown that PAK4 is oncogenic when overexpressed [8] and promotes tumorigenesis *in vivo* [9]. Amplifications of the PAK4 gene have also been identified in pancreatic cancers [10]. In siRNA experiments the loss of PAK4 reduces HGF-dependent cell scattering and migration [11]. The protein is also shown to be required for proper formation of the endothelial lumen [12], consistent with defects seen in PAK4 -/- mice as described [13].

We have shown that Cdc42 directly regulates PAK4 activity in mammalian cells through an auto-inhibitory domain (AID) that binds in a manner similar to pseudo-substrates [14,15]. This is consistent with the notion that PAK4 lacking residues 10–30 in the Cdc42/Rac interactive binding (CRIB) domain is active [16]. Although structural and biochemical analysis suggests that PAK1 activation occurs through activation loop Thr-423 phosphorylation [17], it is notable that PAK4 is constitutively phosphorlyated on Ser-474 [14], and kept in check through the AID. The binding of Cdc42 can serve to activate PAK4 in cells but it is unclear if there is any auto-phosphorylation event associated with this activation [14].

In mammalian cells the role of Cdc42 as a polarity protein has been demonstrated in many contexts, including spindle orientation in mitosis [18]. It is unlikely that in vertebrates the membrane-bound Cdc42 acts at a single ‘polarisome’ as hypothesized in budding yeast [19]. Thus although Cdc42 is Golgi-enriched [1], it is required at cell-cell junctions [20], and has been invoked at the leading edge of cells [21]. Cdc42 is an evolutionarily conserved polarity protein whose effectors include N-WASP, CIP4, IRSp53, TOCA, PAK1 and PAK4 [1,21–25]. Previous reports have also suggested several PAK1 substrates that are common to PAK4 such as LIMK1, Bad and stathmin [26–29]. Although the catalytic domains of the group I and group II PAKs are closely related, they do show some degree of substrate selectivity *in vitro* [30,31]. Cdc42 has been observed to regulate the speed of cell migration [32] and the formation of cell protrusions [33], but often the loss of Cdc42 has no effect on migration speed [34].

In the developing frog embryo, PAK4 (termed X-PAK5) is needed to modulate adherens junction in developing blastomeres [35]. Loss of *Drosophila* PAK4, Mushroom Body Tiny (Mbt), leads to profound defects in the development of the fly brain [36]. Mbt is found at adherens junction and phosphorylates the β-catenin homologue Armadillo [37], thereby weakening cell-cell interactions [38]. PAK4 and Par6 were identified as key effectors in promoting cell-cell junction formation downstream of Cdc42 in bronchial epithelial cells [39]. These are important observations since altered cell polarity is a hallmark of many cancer cells [40,41].

Here we investigate in some detail the localization of PAK4 and the effect of its knockdown. PAK4 is itself localized to cell-cell junctions in the cell lines tested, but is also observed at the centrosome. Although loss of PAK4 was not associated with alteration in collective cell migration, we found that there is a requirement for PAK4 in promoting centrosome reorientation in a monolayer scratch assay. However, its role in promoting cell migration may be cell-type specific. We also identified Ser-675 of β-catenin as a PAK4 kinase target, which is affected upon inhibition of PAK4. Thus, PAK4 is important in establishing cell polarity downstream of Cdc42.

## Materials and Methods

### Antibodies and inhibitors

Primary antibodies used: PAK4 (Proteintech); p120-catenin (Santa Cruz); β-catenin and phospho β-catenin (Ser675) (9562 and 9567, Cell Signaling); Cdc42, tubulin, gamma tubulin, Hoechst and TRITC phalloidin (Sigma); Rac1 and GM-130 (BD transduction laboratories); myosin IIb (Abcam). Alexa488- and 546-conjugated secondary antibodies were from Molecular probes. Horseradish peroxidase (HRP)- conjugated secondary antibodies were from Dako (Carpinteria, CA). The PAK4 inhibitor PF-3758309 (Pfizer) and PKA inhibitor H-89 (Tocris) were used at concentrations of 10μM and 5μM respectively.

### Cell culture and transfection

A few cell lines were used for the experiments. U2OS (ATCC no. HTB-96), COS-7 (ATCC no. CRL-1651), Panc-1 (ATCC no. CRL-1469) and MCF-7 (ATCC no. HTB-22) cells were cultured in DMEM media with 4500mg/L glucose, supplemented with 10% Fetal Bovine Serum (FBS) (Hyclone) and 1% penicillin (100U/ml)-streptomycin (100μg/ml; Invitrogen). LNCaP (ATCC no. CRL-1740) was cultured in RPMI media with similar supplements added to the medium. MDCK (ATCC no. CCL-34) and HeLa (ATCC no. CCL-2) cells were cultured in minimum essential medium (MEM) supplemented with 10% FBS, 2mM L-glutamine, 10mM sodium pyruvate, 0.15% w/v sodium bicarbonate and 0.1mM MEM nonessential amino acids (Invitrogen). All cells were grown at 37°C in an incubator filled with 5% CO_2_ and 99% humidity. For transfection, cells were seeded at 90% confluency and allowed to adhere overnight. Small interfering RNA (siRNA) was transfected (final concentration 50nM) in medium without antibiotics using Lipofectamine 2000 (Invitrogen). Cells were transfected with the required siRNA for 48h before harvesting (for Western analysis) or fixation (for immuno-staining). For DNA transfections, 5ul Lipofectamine 2000 and 1ug plasmid DNA were used for 3X10^5^ cells.

### Cloning and siRNA

Constructs were introduced into pXJ40-based vectors [42] containing N-terminal FLAG- or green fluorescent protein-fusion tags. PAK4 truncation mutants were generated by PCR and point substitutions were generated by QuikChange (Stratagene) mutagenesis. Constructs were confirmed by DNA sequencing. Double stranded siRNAs were purchased from Dharmacon with the following forward sequences: control (AAUUCUCCGAACGUGUCACGU), PAK4 (siPAK4-1) (CCAUGAAGAUGAUUCGGGA) and (siPAK4-2) (GAGAACACUAAGAGGUGAAUU), Cdc42 (GAUUACGACCGCUGAGUUA) and Rac1 (UAAGGAGAUUGGUGCUGUA).

### Wound-healing assay

U2OS cells transfected with the required siRNA were plated to confluency in 35mm dishes the following day and allowed to adhere overnight. The following day, cells were deprived of serum for at least 6h. A cell-free area (wound) was then introduced by scraping the monolayer with a yellow pipette tip, followed by extensive washing to remove the cellular debris and the cells were then replaced in fresh media for 30min to drive cell migration before imaging.

### Immuno-fluorescence and microscopy

Cells were seeded on 22x22mm glass coverslips in serum. The cells were fixed in either 4% paraformaldehyde (vol/vol) at room temperature or methanol at -20°C for 10min depending on the required primary antibody to be used, after which the cells were rinsed and permeabilized in 0.5% TritonX-100 in PBS for 10min. Cells were then blocked with 10% FBS for 10min. Primary antibody diluted in 0.5% TritonX-100 in PBS was added for 2h at room temperature and secondary antibody was diluted similarly and incubated for 1h as well. Coverslips were mounted with fluorescent mounting medium (Thermo Scientific) and visualized using a Roper Scientific CoolSnap HD digital camera adapted to Zeiss Axioplan2 epifluorescent microscope at 63X magnification. Image analyses were performed using Olympus FV10-ASW 1.6 viewer, Image-Pro Plus (Media Cybernetics, Inc, MD) or ImageJ (NIH, USA) software.

### Western blotting

Cell lysates were prepared by scraping cells in protein sample buffer (50mM Tris-HCl pH 6.8, 6% SDS, 100mM DTT, 50% glycerol and 0.1% bromophenol blue) and heated at 95°C for 5min. Proteins were resolved by SDS-PAGE and transferred to PVDF membranes (Immobilon P) at 100V for 2h. These were blocked with 5% BSA in PBS for 1h. Membranes were incubated with primary antibodies (0.25–1.0μg/ml) in 3% BSA/PBS overnight at 4°C, and secondary antibodies for 1h at room temperature. The HRP-conjugated secondary antibodies (Dako) were detected by ECL reagents (GE Healthcare).

## Results

### Endogenous PAK4 localizes to cell-cell junctions in multiple cell types

**Fig 1:**
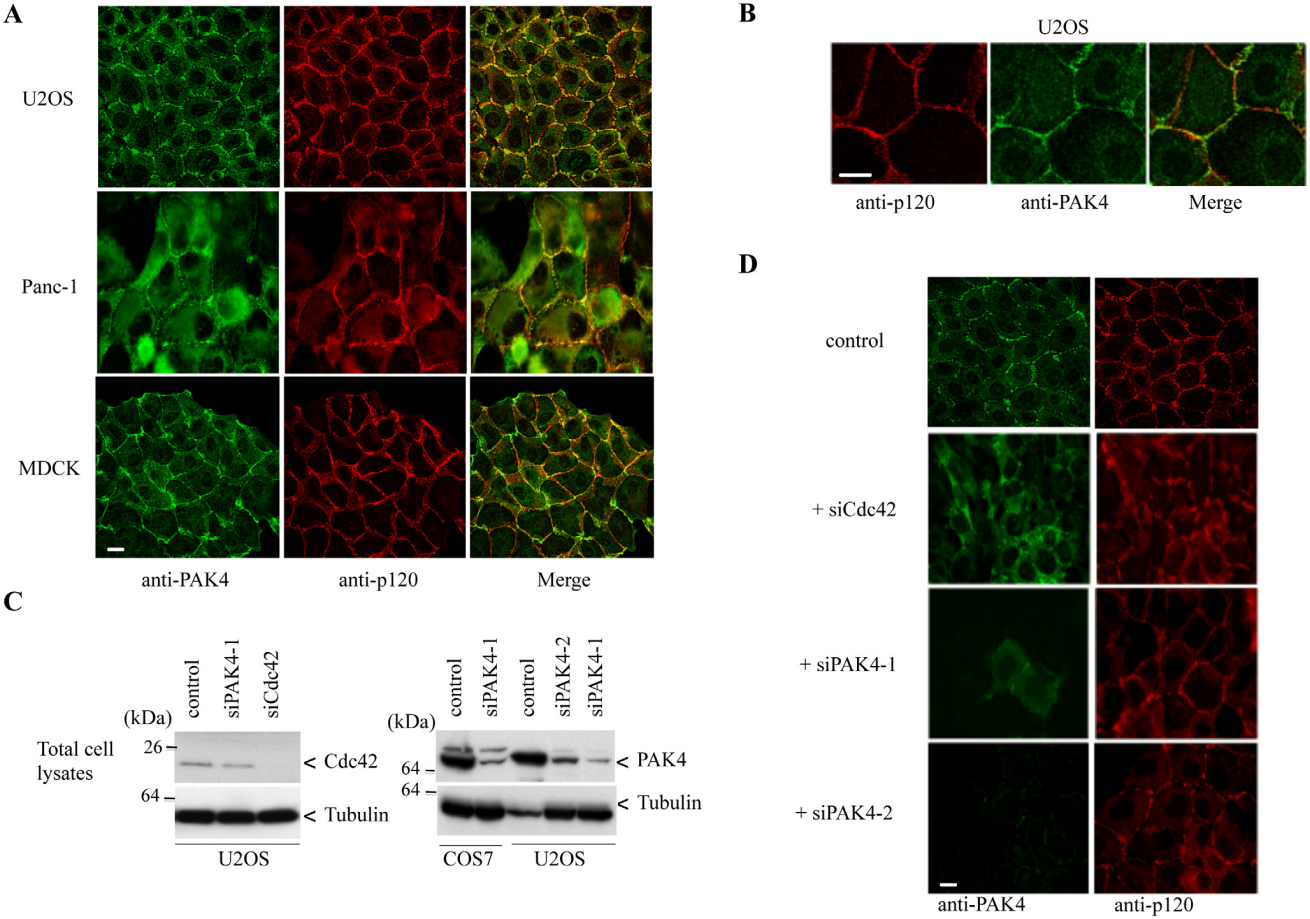
Endogenous PAK4 localization to cell-cell junctions is dependent on Cdc42. A) Cells from different cell lines were plated to confluency onto coverslips and fixed. Fixed cells were immuno-stained with PAK4 and adherens junction marker p120-catenin antibodies. B) Processed cells were imaged under high resolution confocal microscopy to visualize the localization of PAK4 with regard to p120-catenin. C) U2OS or COS-7 cells were transfected with PAK4 or Cdc42 siRNA for 48h and probed for levels of PAK4 and Cdc42 by Western analysis on Triton X-100 soluble cell lysates (30 μg per lane). D) U2OS cells were transfected with Cdc42 siRNA or PAK4 siRNA, fixed after 48h and then immuno-stained for the presence of PAK4 and p120-catenin. Scale bar: 10 μm.

PAK4 is reported to localize to the cytoplasm and the nucleus in mammalian cells [27,39]. When tagged versions of PAK4 were expressed, PAK4 was reported to form a complex with Cdc42 at the Golgi [1] and endogenous PAK4 has been reported to colocalize with ß-COP [43]. PAK4 acts downstream of HGF-treatment and integrin engagement [16,44], but it also likely acts in many contexts in which Cdc42 is activated. In human bronchial epithelial cells PAK4 was located at and was essential for stability of cell-cell junctions [39]. Using a polyclonal anti-PAK4 antibody [14], we observed that PAK4 was strongly enriched at cell-cell junctions in U2OS, Panc-1 and MDCK cells (Fig 1A), and in none of these cells did we find co-staining with focal adhesion markers. The PAK4 signal at cell-cell junctions overlapped but did not colocalize precisely with the adherens junction marker p120-catenin (Fig 1B), which binds to conventional cadherins [45].

### PAK4 binding to cell-cell junctions is dependent on Cdc42

Cdc42 is important for cell-cell junction formation in mammalian epithelial cells [46–48]. In line with its role downstream of Cdc42, the PAK4 *Drosophila* orthologue (Mbt) is needed for proper junction formation in the eye imaginal disk, and mutation of Mbt leads to eye defects [37]. The inhibition of Cdc42 is suggested to prevent adherens junction assembly in MDCK and Caco-2 cells [46–48]. However, other studies have shown that blocking Cdc42 does not significantly affect junction formation in MDCK cells [49,50]. PAK4 was shown to regulate apical junction formation in bronchial epithelial cells; in these cells the kinase was recruited to nascent cell-cell contacts in a Cdc42-dependent manner [39]. Thus, knockdown of Cdc42 leads to loss of cell junctions (including PAK4) in all the three cell types. We tested if the loss of Cdc42 or PAK4 prevents proper junction formation in U2OS cells by treating them with Cdc42 or PAK4 siRNA. As shown in Fig 1C, siRNAs directed to PAK4 or Cdc42 can be extremely effective in reducing levels of the protein at 48h in U2OS (left panel), with an estimated efficiency of knockdown being ~90%, (Western analysis using ImageJ). The PAK4 siRNA was equally efficient in COS-7 cells (Fig 1C, right panel). Knockdown of Cdc42 caused a profound disorganization of cell-cell junctions as assessed by p120-catenin staining (Fig 1D, right panel). The knockdown of PAK4 with two different sets of siRNA however, did not appear to affect cell-cell junctions (Fig 1D, third and fourth row).

### PAK4 knockdown does not affect collective cell migration

**Fig 2:**
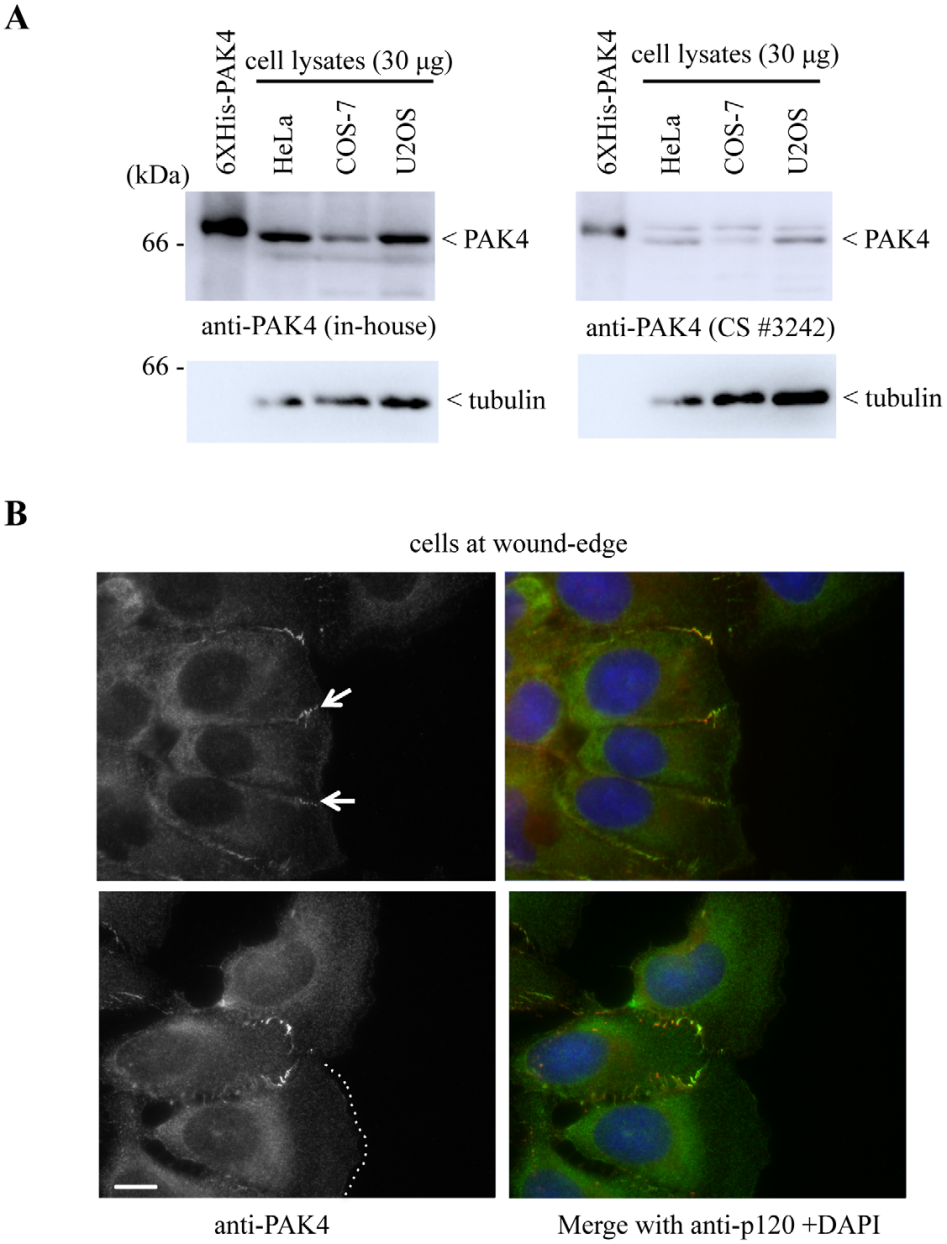
PAK4 does not localize to the leading edge of migrating cells. A) Total lysates (30 μg per lane) extracted from HeLa, COS-7 and U2OS cells were probed for PAK4 by Western blotting using either an affinity purfied PAK4 antibody [14] or Cell Signaling (*3242). Recombinant purified His-tagged PAK4 (6XHis-PAK4) in lane 1 was loaded (10 ng) as a size control. B) Images showing the typical morphology of migrating U2OS cells at the wound-edge in a standard monolayer scratch assay. The cells were fixed with methanol and immuno-stained with anti-PAK4 and anti-p120-catenin. PAK4 enrichment at the cell-cell junction is indicated by white arrows (top panel), and the edge of tone of the lamellipodia is marked with a white dotted line (bottom panel). Scale bar: 5 μm.

The overexpression of PAK4 has been linked to increased cell migration and invasion [51–53], while the knockdown of PAK4 resulted in impaired cell migration in certain cell lines including MDCK cells [54]. It is notable however that other high- and low-throughput studies fail to identify PAK4 siRNA treatment to impact migration rates [55] which could indicate compensation by related kinases. We examined the distribution of PAK4 by indirect immuno-fluorescence in migrating cells using an in-house PAK4 antibody which detects the protein in U2OS, COS-7 and HeLa cells (cf. Fig 2A) as reported previously [14]. The leading edge of U2OS cells undergoing migration exhibit large lamellipodia (cf. dotted line in Fig 2B). In this monolayer scratch format there was no enrichment of PAK4 protein in the cell edge but the kinase was enriched in adherens junctions (as marked by p120 catenin) just back from the cell edge (arrows). Cell junctions at the rear of the cell tend to be lost. It has been reported that PAK4 phosphorylation by PKD1 at Ser-99 is actually required for targeting to the leading edge [56], although these experiments were performed using GFP-PAK4, which does not properly localize to cell-cell junctions (for example in S3A Fig). Endogenous PAK4 is predominantly localized to cell-cell junctions.

**Fig 3:**
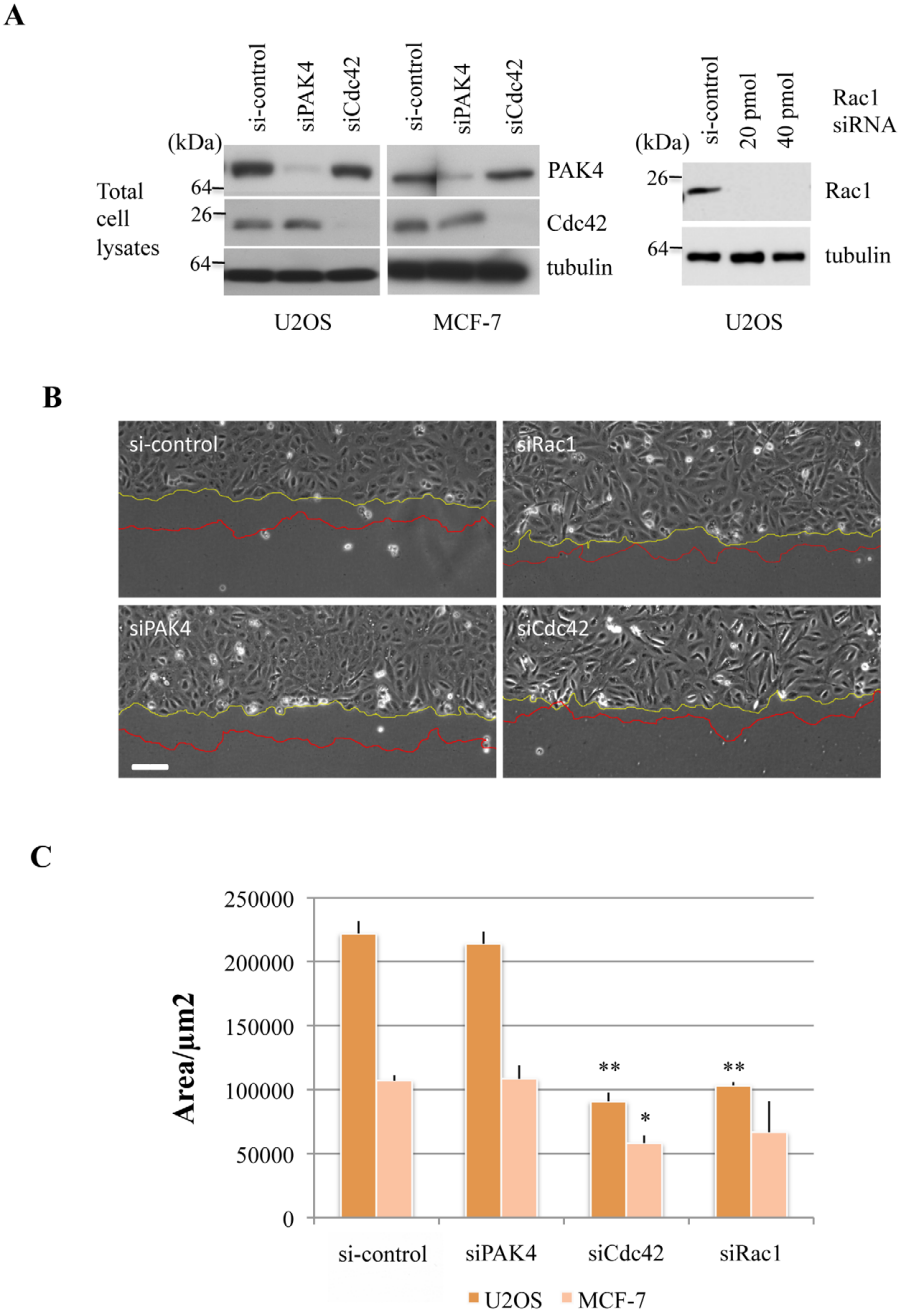
PAK4 loss does not affect collective migration rates of U2OS or MCF-7 cells. A) (Left panel) U2OS or MCF-7 cells were transfected with siRNA directed to PAK4 or Cdc42 as indicated. The cell lysates (30 μg per lane) were probed for expression of PAK4, Cdc42 or tubulin. (Right panel) U2OS cells were transfected with siRNA directed to Rac1 as indicated and the lysates were probed for expression of Rac1 or tubulin. B) Low power images of the same area of the monolayer scratch wound are shown before and after 4h cell migration into the gap. The wound-edge is represented in yellow and red corresponding to the start and end of imaging respectively. C) Bar chart depicting the area covered over 4h after the scratch was applied by either the U2OS or MCF-7 cells, with standard error of mean. The area was calculated using ImageJ software. *P value < 0.05, **P value < 0.005. Scale bar: 50μm.

We tested the effects of PAK4 knockdown in MCF-7 (adenocarcinoma) or U2OS (osteosarcoma) on collective cell migration. The efficacy of knockdown in both cell types was similar (~90% as determined by band signal intensity analysis using ImageJ (Fig 3A). Typical images of leading edge U2OS cells after a monolayer scratch are illustrated in Fig 3B; the position of the monolayer edge at the start and 4h later are marked in yellow and red respectively. The area covered by the migrated cells over this 4h period determined in different regions was averaged over three independent experiments (Fig 3C). Under these conditions, neither U2OS nor MCF-7 cell migration was significantly affected by PAK4 loss. By contrast, knockdown of either Cdc42 or Rac1 caused a significant reduction in migratory rates. We conclude that the role of PAK4 in collective cell migration is cell type and/or growth factor dependent. The U2OS cells treated with PAK4 siRNA also showed no overall changes in the distribution of F-actin or myosin IIB (S1 Fig).

### PAK4 and polarity

In a few cells, we noted that PAK4 sometimes accumulated in a small region often in the vicinity of the nucleus similar to the location of the centrosome (S2A Fig). Disassembling cell-cell junctions by treatment of cells with EGTA, or by plating cells at a low density did not lead to enrichment of PAK4 at the centrosome (S2B Fig), indicating that the cytosolic level of PAK4 is not limiting. We noted that GFP-PAK4 was clearly enriched at the centrosome, as indicated by arrowheads (S3A Fig) but localized poorly to cell-cell junctions. To determine which region of PAK4 is involved in this centrosomal localization, GFP-tagged constructs of PAK4 (as indicated) were co-transfected with RFP-centrin in U2OS cells and analyzed by live cell imaging (S3B Fig). The catalytic domain construct PAK4(300–591) was nuclear enriched with no specific concentration elsewhere. Furthermore, PAK4(300–591) was clearly excluded from the centrosome, as indicated by an asterisk. In contrast both the N-terminal fragments PAK4(1–270) and PAK4(1–64) were centrosome-enriched (marked by arrowheads). Thus, the N-terminal region of PAK4(1–64), which contains the polybasic motif, CRIB and auto-inhibitory domains [14], was sufficient to target GFP-PAK4 to the centrosome. We have noted that GFP-PAK4 localizes poorly to cell-cell junctions, with both N-terminal or C-terminal tags (data not shown), whereas the centrosomal signal appears unaffected.

The role of Cdc42 in polarization is established in many cell types; for example in the polarization of the centrosome in astrocytes, fibroblasts and endothelial cells during directed cell migration [57–60]. This is because Cdc42 is part of a conserved ‘polarity complex’ consisting of Par3, Par6 and PKCζ [61,62]. In order for cells to move the centrosome towards the leading edge, Par6-PKCζ has been suggested to regulate GSK-3β at the leading edge [58,63]. In standard cell monolayer scratch assays, the disruption of the monolayer causes loss of cell-cell contacts and formation of actin-rich protrusions at the leading edge of cells [64]. It is suggested that microtubules emerging from the centrosome and Golgi apparatus are directed specifically toward the cell edge due to dynein pulling forces that preferentially stabilizes them. The displacement of the nucleus towards cell-cell adhesions and away from the free edge also participates in such ‘centrosome reorientation’. This nuclear displacement requires contractile myosin II that is driven through the Cdc42 effector, MRCK [59].

**Fig 4:**
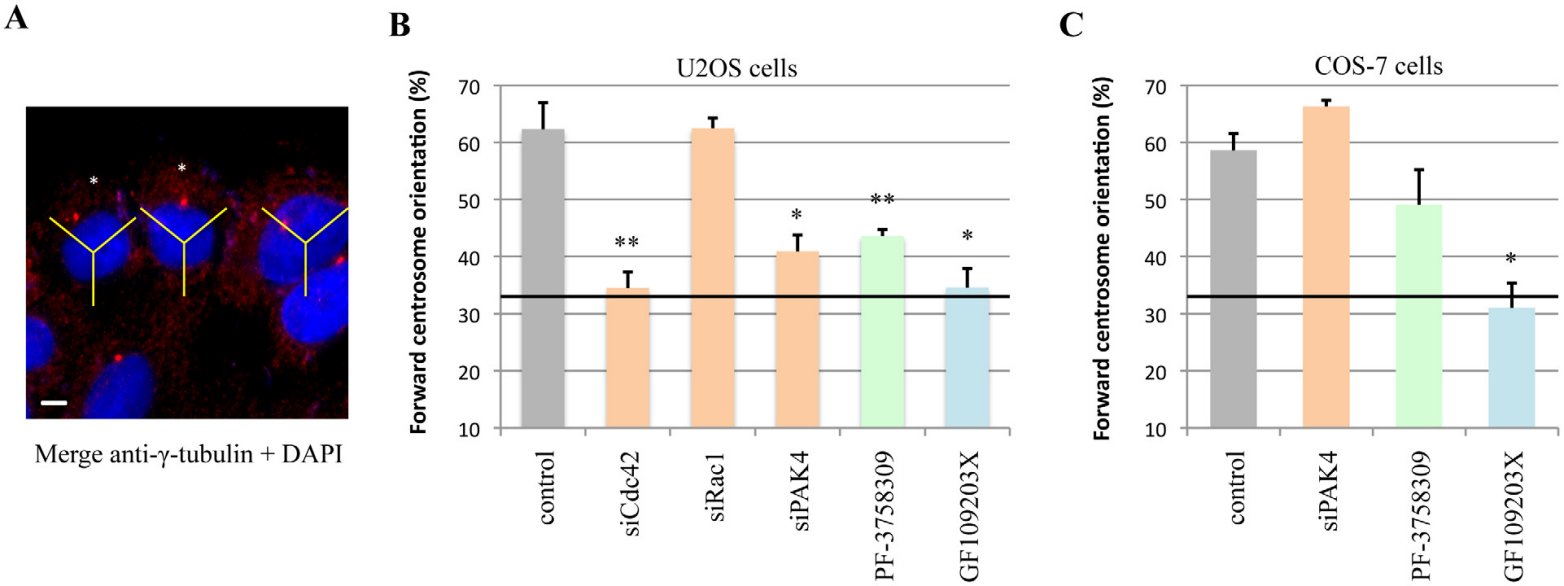
PAK4 regulates centrosomal reorientation in U2OS cells. A) Diagram showing how cells were scored for forward centrosomal reorientation based on immuno-localization of γ-tubulin (prominent red dot) and the nucleus (blue). This was done using a 120° sector (indicated in yellow) centered on the nucleus, and cells with γ-tubulin placed within the forward sector facing the wound edge are scored (as marked with asterisks). The wound is toward the top of the figure and the image was acquired 1h post wounding. B) Graph showing the percentage of U2OS cells with forward centrosome orientation 1h post wounding. Cells were transfected with siRNA to Cdc42, Rac1 or PAK4 and left for 48h before analysis. Cells were also treated with either a PAK4 inhibitor PF-3758309 or a PKC inhibitor GF109203X for 1h prior to scratching. The data represents three independent experiments (N = 60) with standard error of mean. A random orientation of the centrosome gives a 33% baseline (solid black line). C) Graph showing the same analysis performed with COS-7 cells 1h post wounding. Cells were transfected 48h with siPAK4 or treated with inhibitors to PAK4 (PF-3758309) or PKC (GF109203X) for 1h before scratching. *P value < 0.01, **P value < 0.005. Scale bar: 10μm.

To examine the role of PAK4 in centrosome reorientation we knocked-down PAK4 and tested U2OS cells for the centrosome position 1h post-wounding. We checked polarization of the centrosome by scoring their localization in the forward 120° sector as marked by asterisks in Fig 4A. Both control and Rac1 siRNA-treated cells re-orientated their centrosomes normally in response to wounding in the presence of serum (Fig 4B). Compared to the control, PAK4 siRNA-treated cells were almost as severely affected as Cdc42 siRNA-treated cells with respect to polarization, with ~40% of cells showing forward centrosome orientation, which is equivalent to random orientation. This inhibition of polarization occurred when cells were treated with the PAK4 inhibitor, PF-3758309 or with a PKC inhibitor GF109203X [65]. This is in contrast to the lack of effect of PAK4 siRNA on migration rates (Fig 3C). We then tested these effects in COS-7, which we previously showed to show robust centrosome reorientation that is sensitive to Cdc42 loss [66]. Consistent with our previous observations, GF109203X strongly blocked centrosome polarization (Fig 4C). However, neither PAK4 siRNA nor PF-3758309 significantly blocked centrosome reorientation. These experiments indicate that PAK4 is only required for polarization in certain cell types, or that cells with lower levels of PAK4 (Fig 2A) can be compensated by related kinases such as PAK5 and PAK6. Thus, in certain cases PAK4 plays a role alongside other established Cdc42 polarity effectors such as Par6 and MRCK [59] in the reorientation of the centrosome during cell polarization. This data is also consistent with epithelial acini formation being blocked by down-regulating PAK4 [12,67].

### Does PAK4 phosphorylate β-catenin Ser-675?

A role for PAK4 to regulate the dynamics of cell-cell junctions would be consistent with group II PAK kinases arising in metazoans [68]. Cdc42 acts via Par3/Par6/PKCζ complex, which is a major regulator of cell polarity in a variety of cell systems [61,69,70]. Other effector proteins of Cdc42 such as IQGAP1 and N-WASP have also been shown to regulate cell-cell junction formation and maintenance [47,71,72]. PAK4 orthologues in *Drosophila* (Mbt) and *Xenopus* (X-PAK5) are also needed for the proper morphogenesis of cell-cell junctions [35,37]. It has also been shown that *Xenopus* X-PAK5 localizes to adherens junction in animal cap cells and dorsal marginal zone cells, and that kinase inactivity interferes with convergence-extension movements, which require dynamic rearrangements of cell-cell junctions. The PAK4 inhibitor PF-3758309 shows anti-metastatic effect in A549 human lung cancer cells and suppresses β-catenin pathways [73].

**Fig 5:**
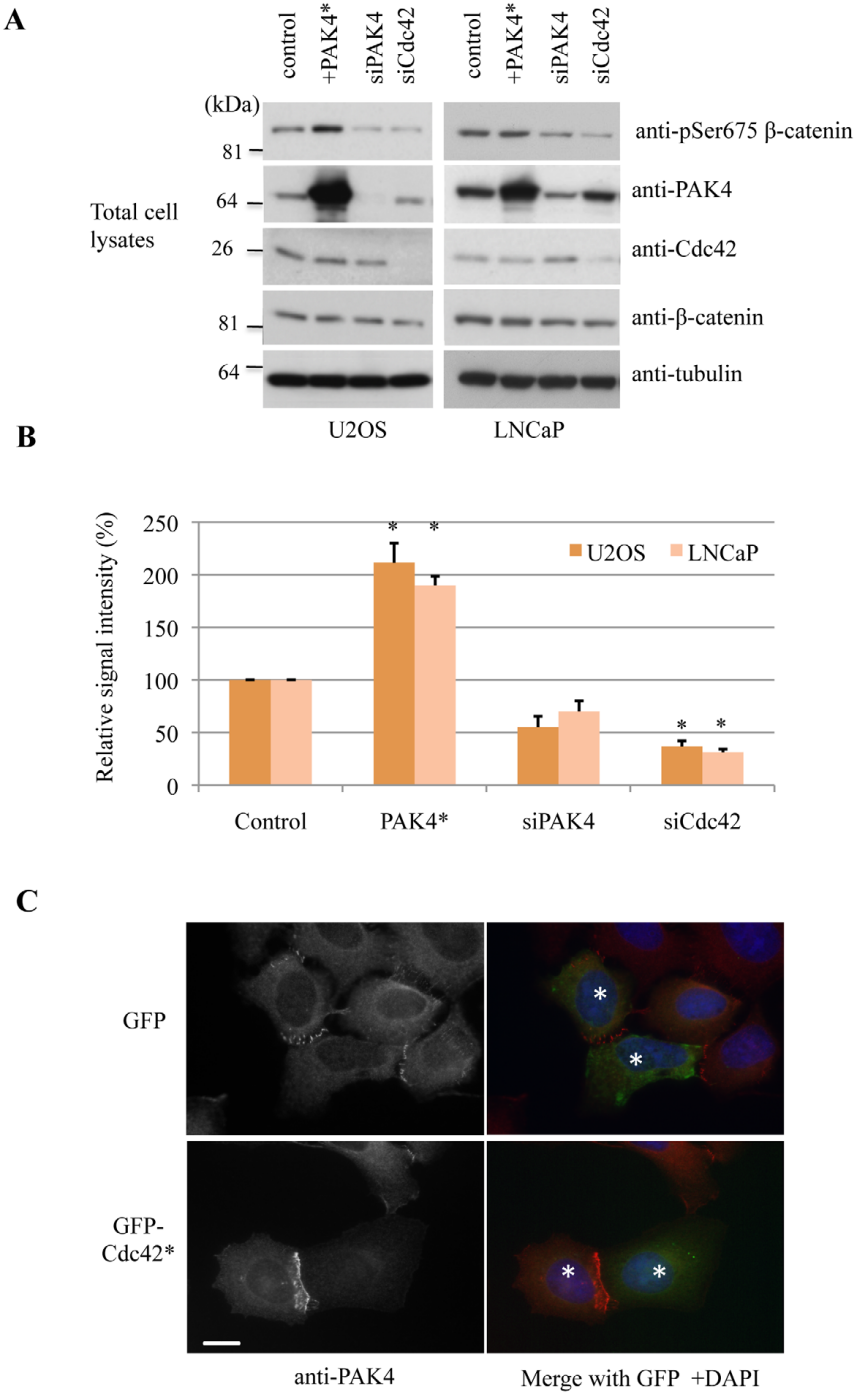
PAK4 phosphorylates β-catenin at Ser-675. A) U2OS or LNCaP cells were transfected overnight with FLAG-PAK4 S445N (denoted PAK4*), or with PAK4 siRNA or Cdc42 siRNA for 48h. The cell lysates (30 μg per lane) processed for Western blotting are as indicated. B) The signal intensities corresponding to bands detected by anti-pSer-675 β-catenin or total anti-β-catenin were obtained from 3 independent experiments using ImageJ, averaged and plotted with standard error of mean. *P value < 0.05. C) U2OS cells were transfected with GFP or GFP-Cdc42(G12V) (GFP-Cdc42*), and fixed the following day. Cells were then immuno-stained for PAK4 and the nuclei were stained by DAPI. Two neighbouring cells in which the cell-cell junction was observed are denoted by asterisks. Scale bar: 10μm.

**Fig 6:**
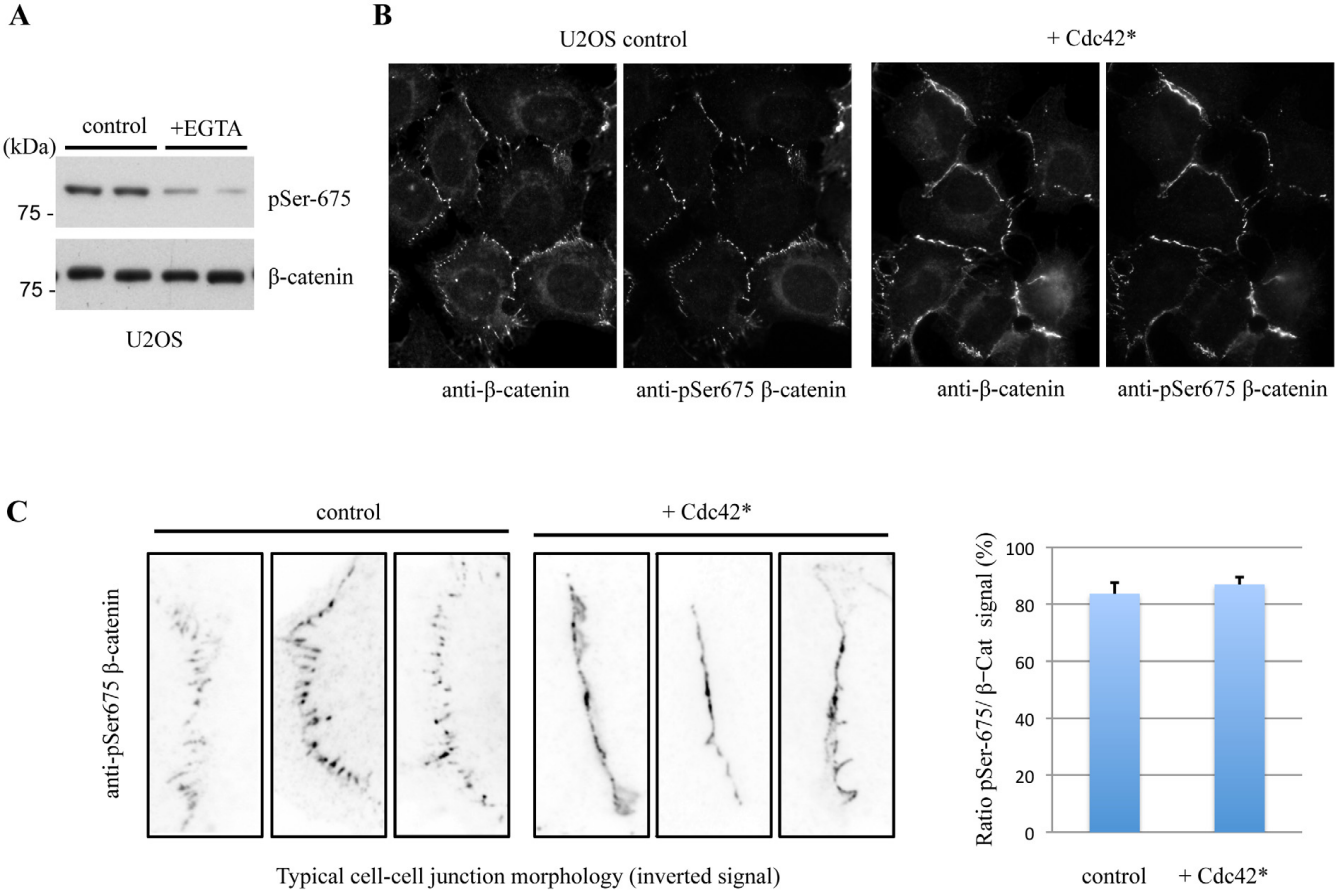
Active Cdc42(G12V) alters the morphology of cell-cell junctions. A) U2OS cells were treated with 4mM EGTA in normal media for 30min to allow cell junction disassembly (see S2 Fig); the cell lysates (30 μg per lane) processed for Western blotting are as indicated. B) U2OS cells were transfected with FLAG-Cdc42(G12V) (denoted as Cdc42*) and fixed the following day. Cells were then immuno-stained for pSer-675 β-catenin and total β-catenin and a representative field is shown. C) The typical cell-cell junction morphologies of controls or FLAG-Cdc42(G12V)-transfected cells are shown for pSer-675 β-catenin. Total signal intensities of junctional pSer-675 β-catenin was summed and likewise, total β-catenin. The average ratio for the two signals for (8 junctions) is shown on the right side, with standard error of mean.

Since PAK4 partially colocalizes with adherens junctions (marked by p120-catenin in Fig 1B) and has been reported to phosphorylate β-catenin both in mammalian cells and in *Drosophila* [38,74], we decided to look at this aspect in more detail. Other studies report Akt and PKA as the prominent β-catenin Ser-552 and Ser-675 kinases respectively [75,76]. There is also strong evidence that Ser-675 is modified by PAK1 in breast epithelial cells [77]. The phosphorylation of β-catenin Ser-675 may enhance its transcriptional activity [78], although the role for this modification at cell-cell junctions is unknown. Since MCF-7 cells have lower levels of β-catenin, we used U2OS and LNCaP cells (Fig 5A), which readily form cell-cell junctions. Active PAK4 (PAK4 S445N, denoted as PAK4*) was used as a positive control to confirm that the active kinase (when overexpressed) acts on β-catenin. While active PAK4 increased pSer-675 levels, the knockdown of PAK4 did not reduce β-catenin pSer-675 levels within statistical significance (Fig 5B), when normalized to total β-catenin levels to account for indirect influences on β-catenin stability [79,80]. When cells were treated with EGTA to disassemble cell-cell junctions, the phosphorylation of β-catenin was strongly inhibited as assessed by pSer-675 levels (Fig 6A).

Cdc42 knockdown by siRNA leads to loss of junctions in many cells including U2OS (Fig 1), which will affect a gamut of junctional proteins. U2OS cells transfected with GFP-tagged constitutively active Cdc42(G12V) (denoted by Cdc42*) showed enhanced PAK4 localization to cell-cell junctions (Fig 5C) and enhanced localization of β-catenin to the cell-cell junctions. It was also notable that these junctions had a more 'mature' morphology (Fig 6B). This change in morphology was not found in the control cells at this point after transfection and plating. We then calculated the ratio of signal due to pSer-675 versus total β-catenin (using a monoclonal antibody directed to β-catenin) in Cdc42(G12V)-transfected cells (Fig 6C). In transfected cells, the pSer-675 signal was as sensitive to PF-3758309 as control cells (data not shown). It is likely that Cdc42 activation by various RhoGEFs [47], drives junctional assembly which is accompanied by PAK4 recruitment and modification of β-catenin. When we drive 'mature' junctions by ectopic Cdc42(G12V), the proportion of β-catenin phosphorylated at pSer-675 remains the same.

**Fig 7:**
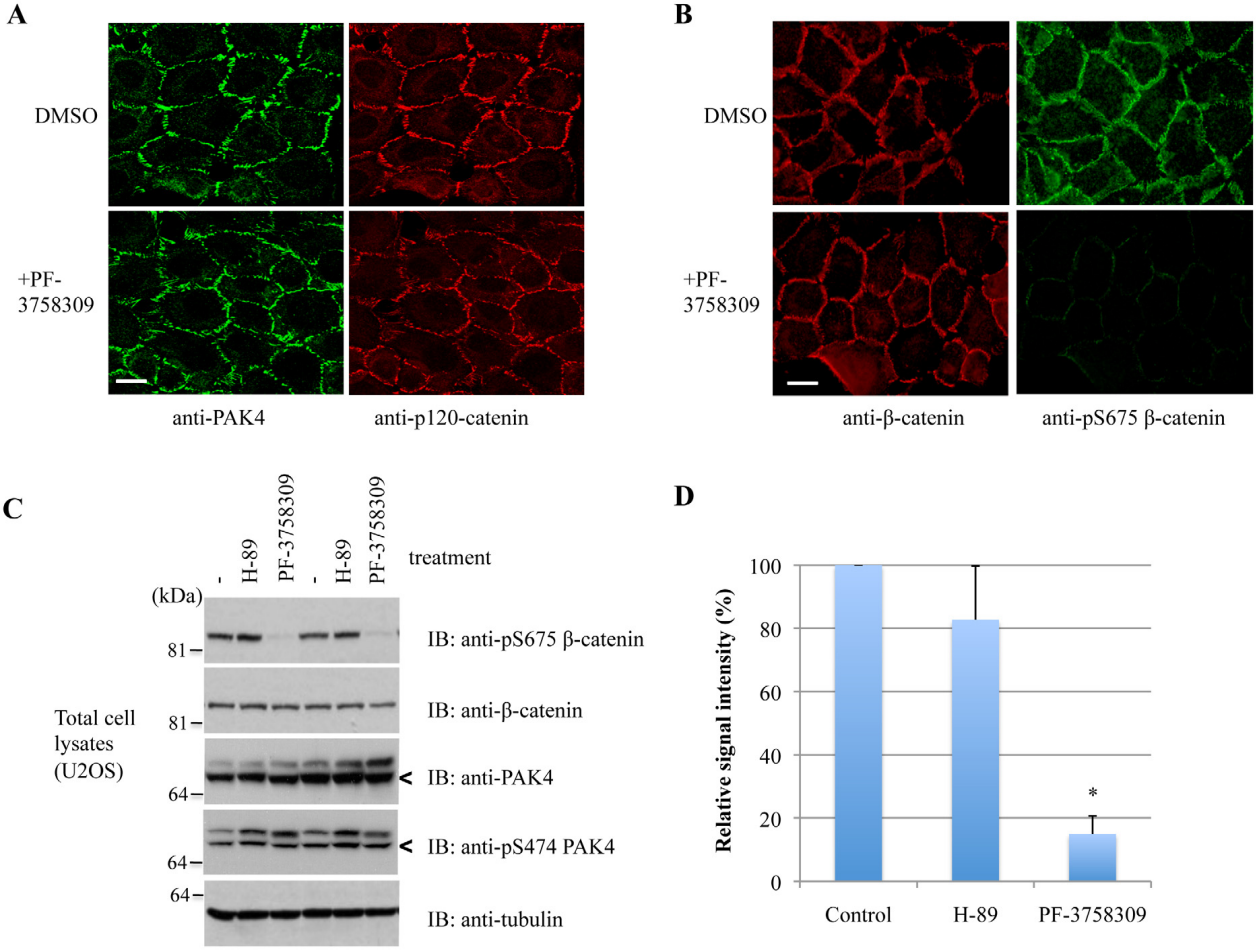
Inhibition of PAK4 affects β-catenin Ser-675 phosphorylation. A) U2OS cells were treated with an inhibitor to PAK4 (5 μM PF-3758309) or vehicle for 1h before fixation, and then immuno-stained for PAK4 and p120-catenin. B) U2OS cells were similarly treated as in (A), but immuno-stained for pSer-675 β-catenin and p120-catenin instead. C) U2OS cells were treated with an inhibitor to PKA (10 μM H-89) or 5 μM PF-3758309 for 1h before lysates were harvested and subjected to Western blotting. The PAK4 bands are marked by arrowheads. D) Band signal intensities of β-catenin pSer-675 against total β-catenin was obtained from Western blots in 3 independent experiments via analysis using ImageJ, averaged and plotted with standard error of mean. *P value < 0.05. Scale bar: 10μm.

To confirm β-catenin Ser-675 is a target of PAK4, cells were treated for 1h with PF-3758309, a PAK4 inhibitor [81] that does not reduce PAK4 staining at cell-cell junctions (Fig 7A). We consistently observed a profound loss of pSer-675 β-catenin signal from cell-cell junctions (Fig 7B). The inhibitor was more penetrant than that of PAK4 siRNA (Fig 5B) because the effect is uniform across cells while siRNA requires PAK4 degradation. Treatment with PF-3758309 did not change β-catenin junctional disposition, nor was any of the pSer-675 signal associated with the nucleus, as previously reported [74]. Short-term treatment with PF-3758309 strongly attenuated pSer-675 levels by Western analysis (Fig 7C and 7D) but PKA inhibition (by H-89), which is reported as a Ser675 kinase [76], had no effect. Since in this context PAK1 was reported to phosphorylate the same target site [82], we examined pSer-675 phosphorylation with expression of the pan-Group I PAK autoinhibitory domain (PAK2-autoinhibitory domain, AID2). The pSer-675 signal at junctions in AID2-expressing cells was indistinguishable from controls (S4 Fig). We conclude that under these conditions, PAK4 rather than PAK1/2 acts on β-catenin, which is consistent with PAK4 being activated by Cdc42 at cell-cell junctions.

**Fig 8:**
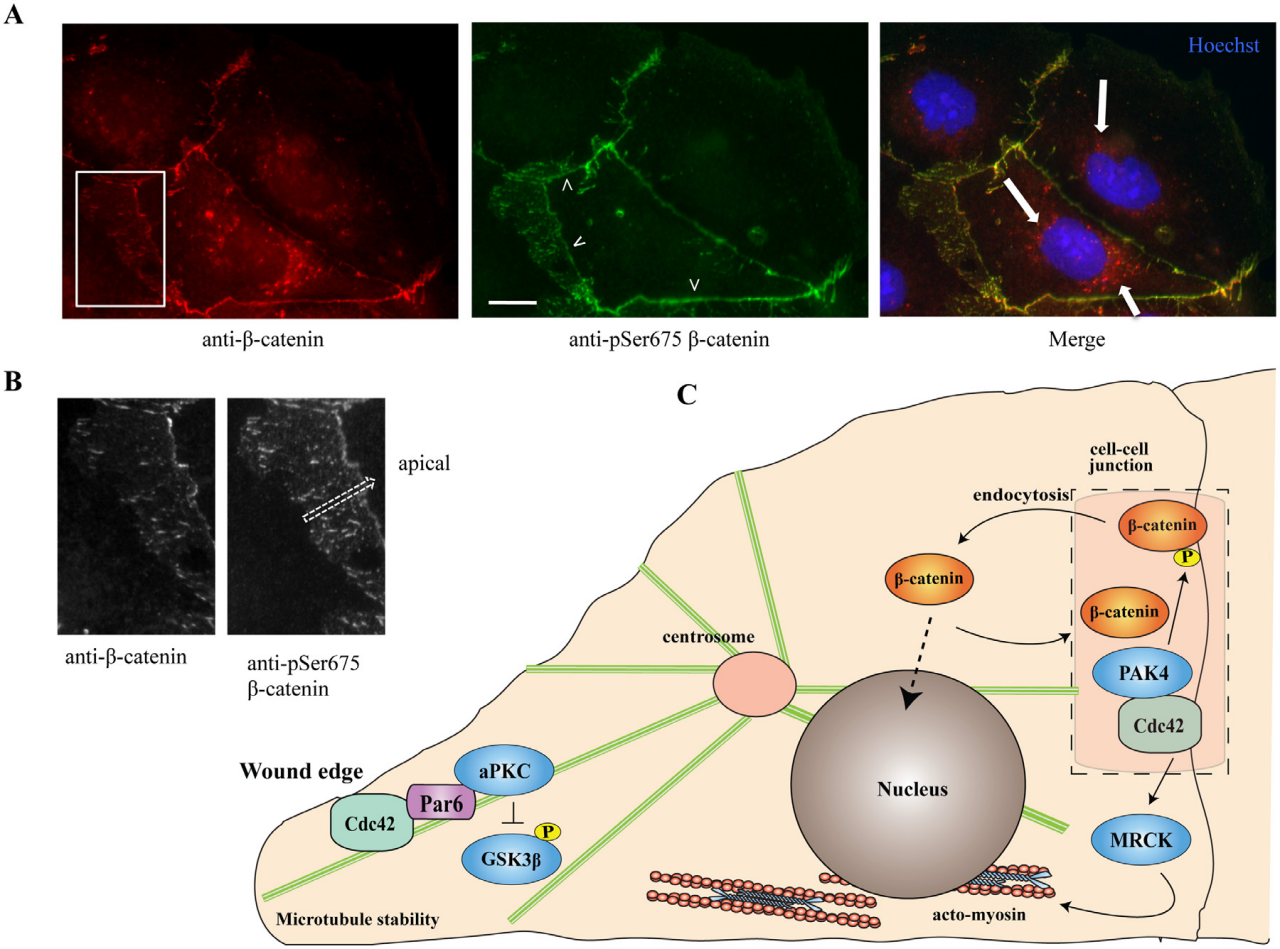
Analysis of the site of β-catenin phosphorylation, and a model for Cdc42 effector function in centrosome reorientation. A) Caco-2 cells were fixed and immuno-stained with anti-pSer675 and anti-β-catenin and nuclei stained during mounting with Hoechst. The white arrowheads in the middle panel indicate phophorylated β-catenin at the typical band-like zona adherance (ZA) but the signal is notably absent from the nucleus. The arrows in the right panel indicate consistent presence of non-phosphorylated β-catenin enriched on what appear to be vesicles in the peri-nuclear region. Scale bar: 10μm B) A magnified view of the boxed area in Fig 8A. The cadherin containing clusters distribute from the base of the cells, that is where cells attach to the extracellular matrix, apically to the region where the unbroken radial ZA is present. The pSer-675 modified β-catenin is detected across the whole region (marked by arrow). C) A model for the role of Cdc42 in coordinating cell polarity during nuclear/centrosome reorientation. The presence of Par6-PKCζ at the leading edge of cells has been suggested to regulate GSK-3β [63]. The activity of GSK-3β can affect many proteins and prominently negatively regulates those involved with microtubule stabilization [99]. A functional microtubule network is necessary for centrosomal positioning, such as the interaction of dynein with microtubules. PAK4, which is found at cell-cell junctions, phosphorylates β-catenin on Ser-675; this modification is absent from intracellular β-catenin, and does not appear to be involved in its nuclear accumulation. The kinase MRCK acts on contractile myosin II to promote nuclear movement.

Our data are consistent with local phosphorylation of β-catenin on Ser-675 by PAK4 at cell-cell junctions (Fig 8A). We looked at human Caco-2 cells which contain small cadherin complexes (puncta) that assemble near the basal surface [83] in addition to a continuous band of the zonula adherens. The non-phosphorylated β-catenin was found to be enriched in vesicles near the peri-nuclear region, likely to represent a pool bound to cadherins that undergo recycling. Thus β-catenin internalization is probably associated with its dephosphorylation. Based on the pSer-675 signal, we propose that this modification of β-catenin takes place in regions where active PAK4 (bound to Cdc42) overlaps with β-catenin at cell-cell junctions (Fig 8B), but does not occur on internalized vesicles where we do not observe PAK4.

## Discussion

The generation of cell polarity occurs in many contexts and often involves a conserved molecular machinery centred around the small GTP-binding protein Cdc42 [84,85]. In metazoans, this process has been shown to require the spatially-restricted activation of Par3/Par6/aPKC complex [57], where Par6 interacts directly with Cdc42.GTP [86]. Since PAK4 is also an effector of Cdc42 whose interaction elevates kinase activity *in vivo* [14], we hypothesize that PAK4 activity is important for cell polarization. Based on our findings regarding the immuno-localization of PAK4 to cell-cell junctions in multiple cell types (Fig 1), it seems reasonable to propose that PAK4 activity downstream of Cdc42 [39] is responsible in establishing certain types of cell polarity. While the knockdown of Cdc42 often disrupts cell-cell junctions [39,87], we did not find that loss of PAK4 affected these structures (Fig 1D).

Unlike PAK1 which localizes to the leading edge of migrating cells, we did not find PAK4 to be enriched in this region (Fig 2B). Indeed PAK4 knockdown in our hands failed to grossly affect the actin cytoskeleton or migration (S1 Fig and Fig 3). PAK4-null mouse embryos [6] primarily exhibit defects in the heart and brain, which are not specifically related to cell migration. One PAK4 substrate which is found at cell-cell junctions is Par6b [88]. Two other PAK4 associated proteins Gab1 and DGCR6L are signaling elements that could act as PAK4 adaptors [54,89].

Cdc42 is needed to promote centrosome reorientation in multiple cell types including U2OS cells (Fig 4B). In this pathway, we anticipate that Cdc42 acts on multiple effectors including Par6/PKCζ (as illustrated in Fig 8C), which is reported to stabilize microtubule plus ends in the direction of cell movement [90]. APC and Dlg1 also play a role in cell polarization by affecting centrosome reorientation [91]. Phosphorylated β-catenin may be found in centrosomes, and loss of the protein strongly interfers with cell polarity and neurogenesis in the developing midbrain [92]. However, studies like this have focused on the phosphorylation of Ser-33/Ser-34/Thr-41, which regulates β-catenin turnover while little is known about the role of Ser-675 modification.

Cdc42 and MRCK are also implicated in nuclear movement via effects on the sub-nuclear actomyosin network [59]. Taken together, it is likely that PAK4 acts downstream of Cdc42.GTP at cell-cell junctions where it accumulates along with other junctional effectors such as N-WASP and Par6 [39]. The other kinase effector MRCK is located at cell-cell junctions through an interaction with ZO-1 [93] as well as with myosin II and myosin18A in subnuclear acto-myosin fibres [94]. A model to summarize the role of these various Cdc42 effectors is illustrated in Fig 8C.

Our data in several mammalian cell types indicates that PAK4 is localized to the cell-cell junctions and the centrosome (Fig 1 and S3 Fig). While the centrosomal localization has been localized to PAK4 residues 1–64, the requirement of cell-cell junction targeting is not clear because GFP-tagged PAK4 localizes poorly compared with the untagged protein. The formation of epithelial junctions (with tight and adherens junction domains) requires the Cdc42 target Par6 and atypical PKCζ [95,96]. It is likely that the heart and brain defects seen in PAK4 knockout mouse embryos originate from abnormal cell-cell junction signalling [6]. In *Drosophila*, the PAK4 orthologue Mbt [97] was suggested to target Armadillo (homologue of β-catenin), and deletion of Mbt from the eye leads to abnormal cell-cell junctions [38]. Our evidence (Fig 7) is that the inhibition of PAK4 by PF-3758309 strongly suppressed phosphorylation of the β-catenin Ser-675 site. The role of β-catenin phosphorylation at this site has been suggested as controlling nuclear-cytoplasmic dynamics [74]. However, immuno-staining experiments showed that the anti-pSer-675 signal remains associated with cell-cell junctions and with regard to S4 Fig, the kinase responsible for the phosphorylation is likely to be PAK4 instead of PAK1/2.

The identification of PAK4 as a kinase that is directly regulated by Cdc42 [14] suggests that the kinase is activated at cell-cell junctions where Cdc42 acts on other targets such as Par6b. But the exact role of this metazoan-specific kinase remains challenging in the light of recent data that loss of PAK4 in fish yields no phenotype [98], although loss of the *Drosophila* PAK4 orthologue Mbt yields strong nervous system defects [37]. Our data provides evidence for PAK4 acting downstream of Cdc42 in pathways required for cell polarization (Fig 8C), in which other effectors such as Par6 and MRCK have also been implicated [57,59]. Further experiments are under way to establish the partners of PAK4 at cell-cell junctions and how these contribute to such targeting.

## Supporting Information

S1 Fig PAK4 knockdown does not affect F-actin. (Top panels) U2OS cells (control) or treated with PAK4 siRNA were fixed with 4% paraformaldehyde 48h after transfection and immuno-stained with anti-PAK4 and TRITC-phalloidin. (Bottom panels) U2OS cells were also fixed in methanol and immuno-stained with anti-PAK4 and anti-myosin IIB antibodies. Scale bar: 10μm.
(TIF)

S2 Fig Disassmbly of cell-cell junctions by treatment with EGTA or re-plating does not lead to enrichment of PAK4 at the centrosome. A) U2OS cells treated with Cdc42 siRNA were immuno-stained for PAK4 and imaged. Images show PAK4 localization at the centrosome in different cells (circled in white). B) U2OS cells were treated with 4mM EGTA for 30min before fixation and immuno-staining with PAK4 and γ-tubulin antibodies (middle row). Cells were also plated at low density and similarly immuno-stained (bottom row). Scale bar: 10μm.
(TIF)

S3 Fig PAK4 localizes to the centrosome in a Cdc42-independent manner via the N-terminus. A) U2OS cells transfected with GFP-PAK4 were fixed with methanol and immuno-stained with γ-Tubulin. PAK4 localization at the centrosome is indicated with white arrowheads. B) U2OS cells were transfected with GFP-PAK4 deletion constructs together with RFP-centrin as a centrosomal marker and imaged under live confocal microscopy. PAK4 localization at the centrosome is indicated with arrowheads. Exclusion from the centrosome for PAK4(300–591) is indicated with an asterisk. Scale bar: 5μm.
(TIF)

S4 Fig Inhibition of group I PAKs does not affect β-catenin Ser-675 phosphorylation. U2OS cells were transfected with GST-tagged PAK2 autoinhibitory domain (GST-AID2). Cells were then immuno-stained for pSer-675 β-catenin, GST and Hoechst. The pSer-675 β-catenin signal at junctions in AID2-expressing cells was indistinguishable from controls. Scale bar: 10 μm.
(TIF)
