# Supplementary figures and images for: The Cdc42 Effector Kinase PAK4 Localizes to Cell-Cell Junctions and Contributes to Establishing Cell Polarity
Source: PLoS One. 2015 Jun 11;10(6):e0129634. doi: 10.1371/journal.pone.0129634 (PMC4466050; doi:10.1371/journal.pone.0129634)

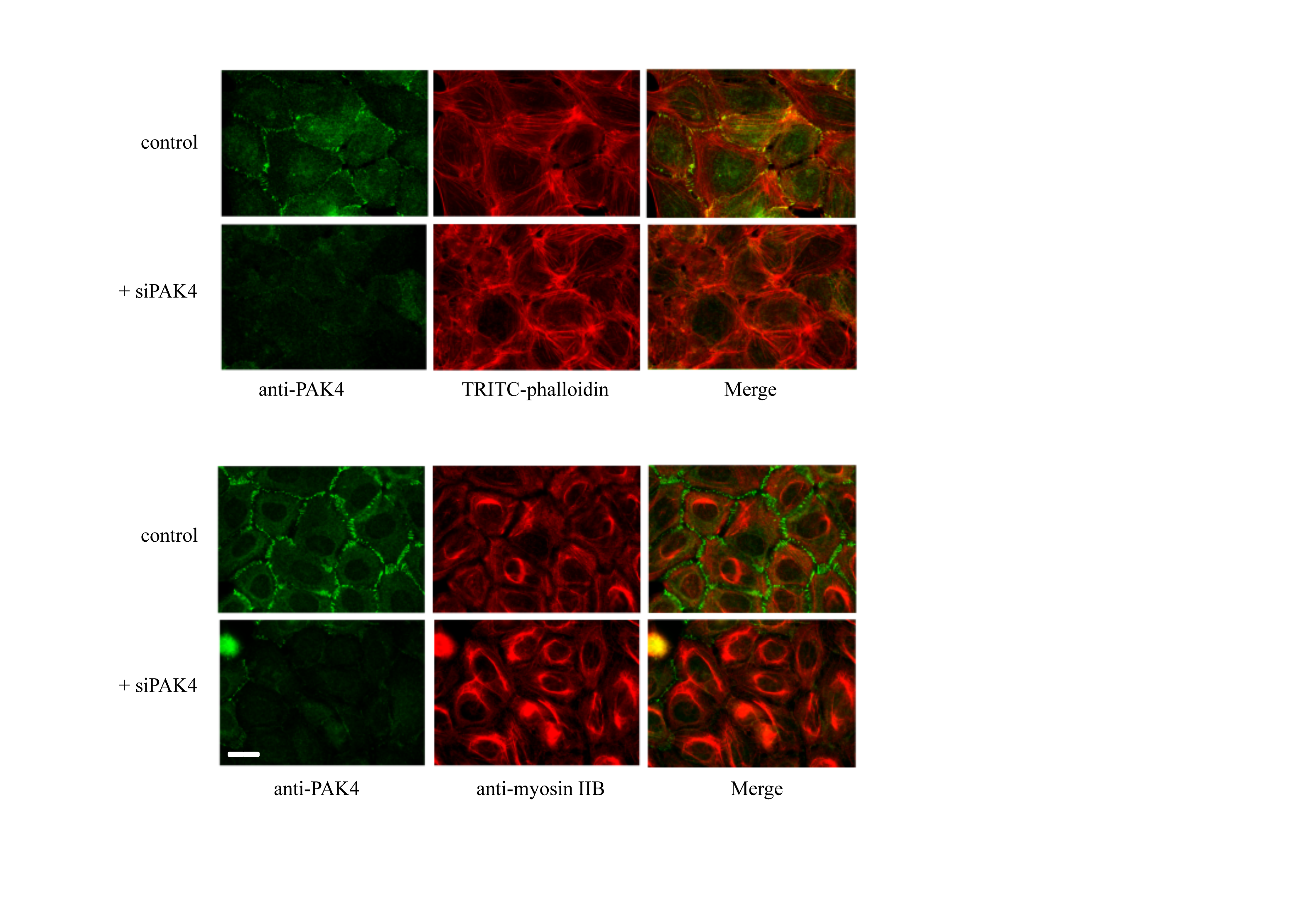

Supplement: S1 Fig — (Top panels) U2OS cells (control) or treated with PAK4 siRNA were fixed with 4% paraformaldehyde 48h after transfection and immuno-stained with anti-PAK4 and TRITC-phalloidin. (Bottom panels) U2OS cells were also fixed in methanol and immuno-stained with anti-PAK4 and anti-myosin IIB antibodies. Scale bar: 10μm. (TIF) [file pone.0129634.s001.tif]

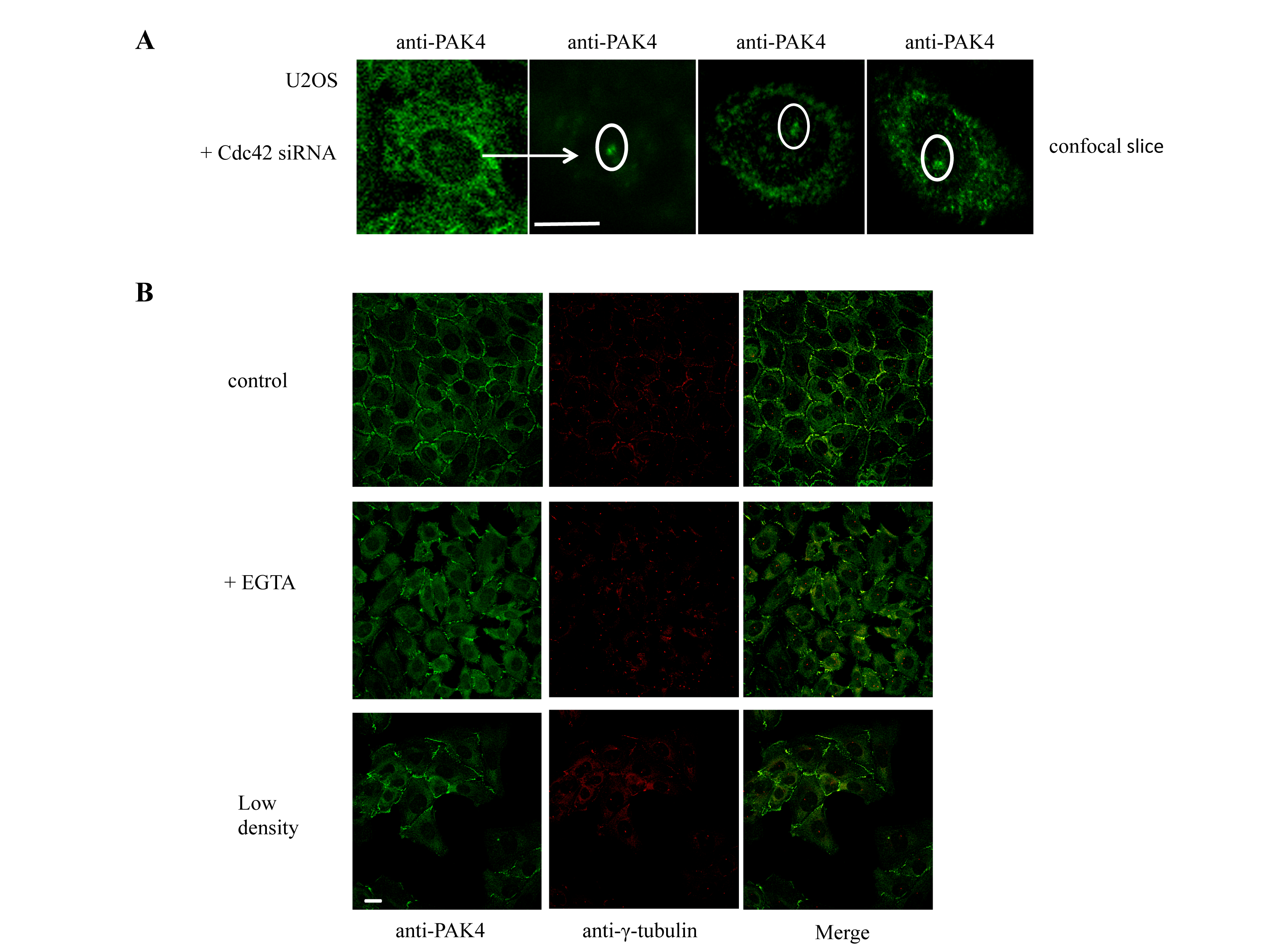

Supplement: S2 Fig — A) U2OS cells treated with Cdc42 siRNA were immuno-stained for PAK4 and imaged. Images show PAK4 localization at the centrosome in different cells (circled in white). B) U2OS cells were treated with 4mM EGTA for 30min before fixation and immuno-staining with PAK4 and γ-tubulin antibodies (middle row). Cells were also plated at low density and similarly immuno-stained (bottom row). Scale bar: 10μm. (TIF) [file pone.0129634.s002.tif]

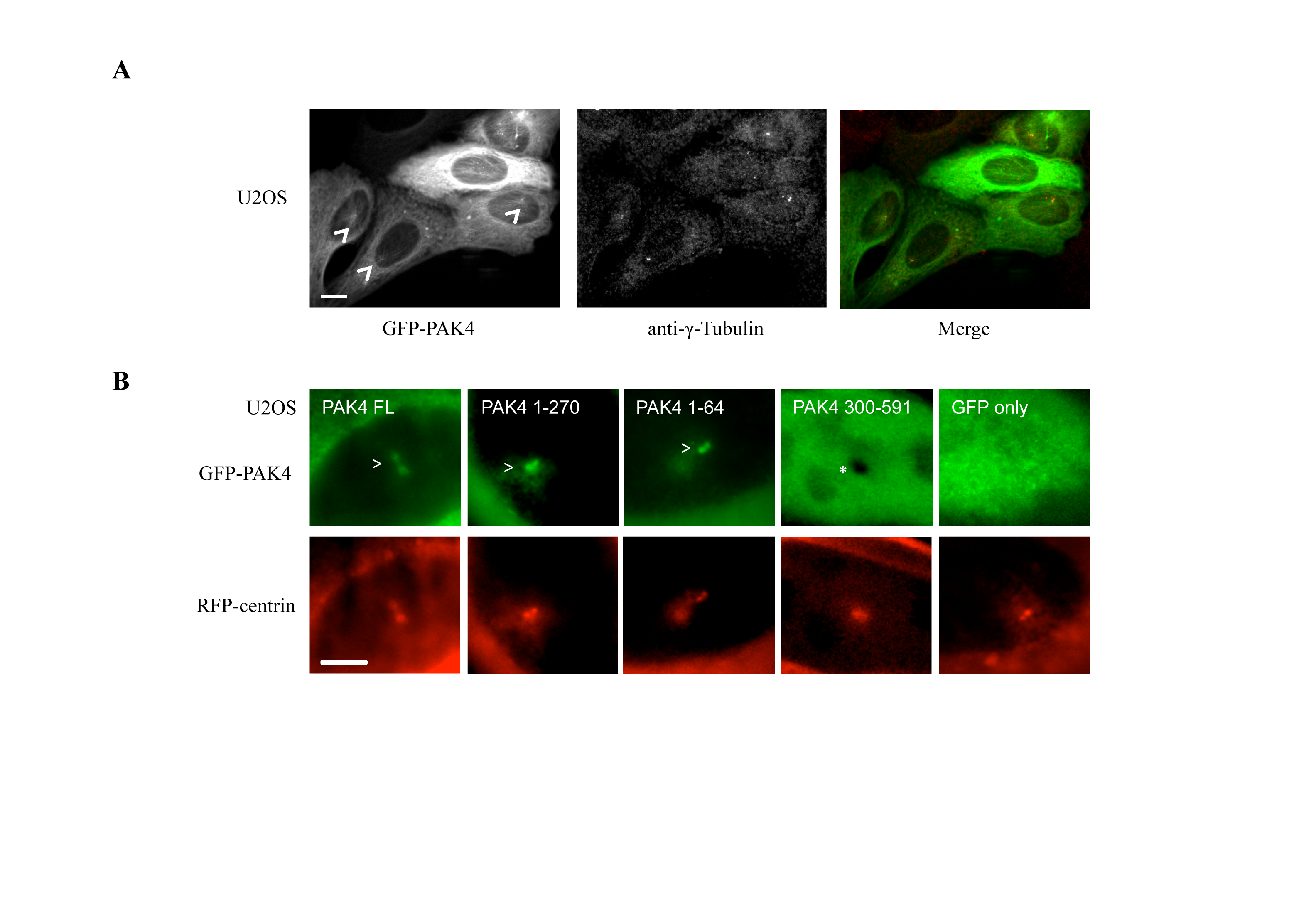

Supplement: S3 Fig — A) U2OS cells transfected with GFP-PAK4 were fixed with methanol and immuno-stained with γ-Tubulin. PAK4 localization at the centrosome is indicated with white arrowheads. B) U2OS cells were transfected with GFP-PAK4 deletion constructs together with RFP-centrin as a centrosomal marker and imaged under live confocal microscopy. PAK4 localization at the centrosome is indicated with arrowheads. Exclusion from the centrosome for PAK4(300–591) is indicated with an asterisk. Scale bar: 5μm. (TIF) [file pone.0129634.s003.tif]

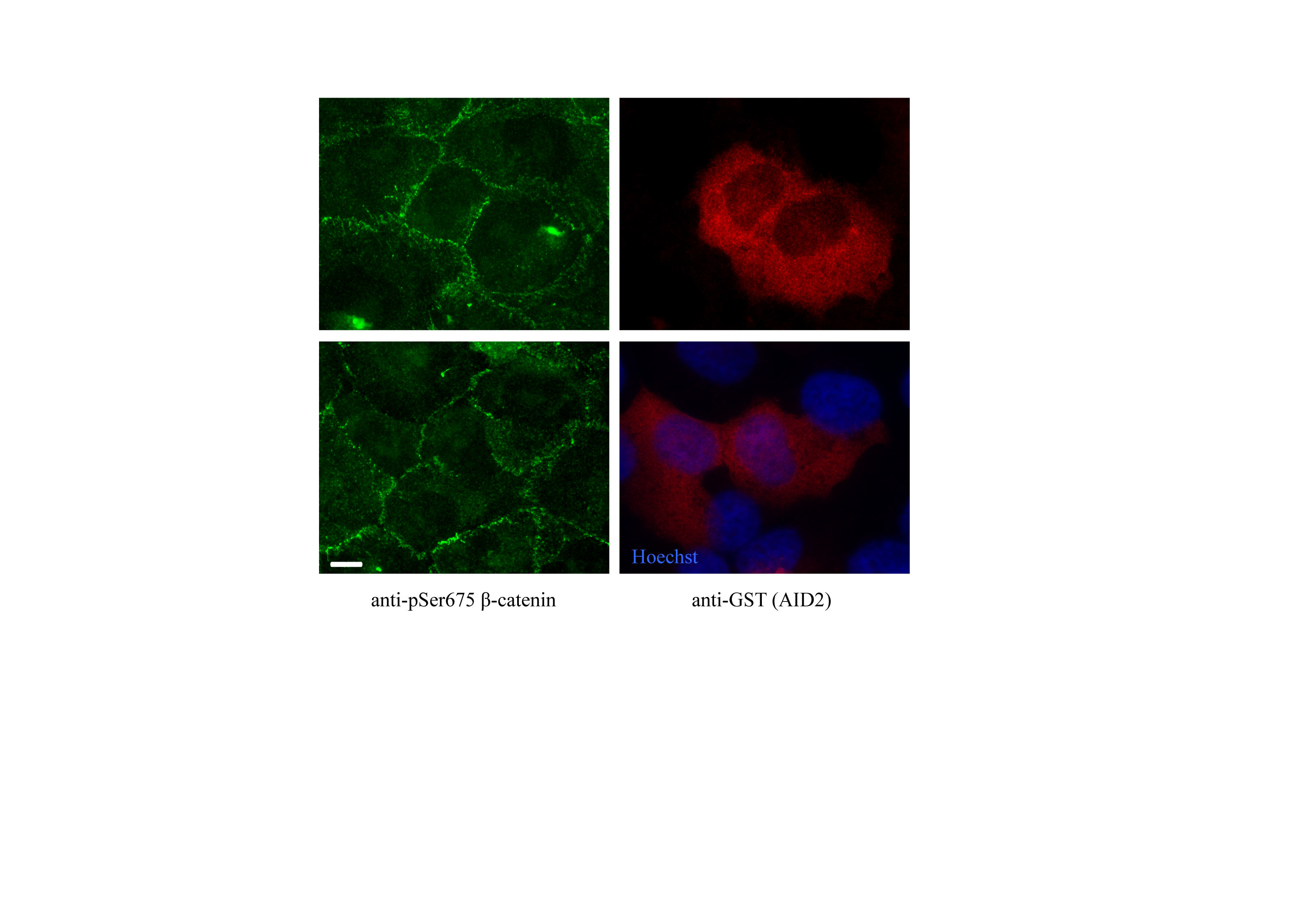

Supplement: S4 Fig — U2OS cells were transfected with GST-tagged PAK2 autoinhibitory domain (GST-AID2). Cells were then immuno-stained for pSer-675 β-catenin, GST and Hoechst. The pSer-675 β-catenin signal at junctions in AID2-expressing cells was indistinguishable from controls. Scale bar: 10 μm. (TIF) [file pone.0129634.s004.tif]
